# Water Decontamination with Magnetic Particles by Adsorption and Chemical Degradation. Influence of the Manufacturing Parameters

**DOI:** 10.3390/ma13102219

**Published:** 2020-05-12

**Authors:** Paulo A Augusto, Teresa Castelo-Grande, Diana Vargas, Lorenzo Hernández, Leticia Merchán, Angel M Estevez, Juan Gómez, José M Compaña, Domingos Barbosa

**Affiliations:** 1 Departamento de Ingeniería Química y Textil, Facultad de Ciencias Quimicas, Universidad de Salamanca, Plaza de los Caídos 1-5, 37008 Salamanca, Spain; dmariavsp@gmail.com (D.V.); loherga@usal.es (L.H.); leticiamerchan@usal.es (L.M.); estevez@usal.es (A.M.E.); 2 LEPABE—Laboratory for Process Engineering, Environment, Biotechnology and Energy, Faculty of Engineering, University of Porto, Rua Dr. Roberto Frias, s/n, 4200-465 Porto, Portugal; tcg@fe.up.pt (T.C.-G.); dbarbosa@fe.up.pt (D.B.); 3 Departamento de Geología, Facultad de Ciencias, Universidad de Salamanca, Pza. de Los Caídos s/n, 37008 Salamanca, Spain; jugb@usal.es; 4 Servicio de Difracción de Rayos-X, Universidad de Salamanca, Pza. de Los Caídos s/n, 37008 Salamanca, Spain; jmcompana@usal.es

**Keywords:** magnetic particles, synthesis, optimized values, large-scale operations, COD removal, wastewater treatment, colorant degradation, sorption, Fenton reaction

## Abstract

Many different processes for manufacturing of magnetic particles are present in scientific literature. However, the large majority are not able to be applied to large-scale real operations. In this study, we present an experiment undertaken to determine advisable values and options for the main variables and factors for the application of the reverse co-precipitation method to produce magnetic particles for real environmental applications. In such, we have tried a conjugation of values/factors that has led to 12 main experiments and production of 12 different particles. After an initial study concerning their main characteristics, these 12 different particles were applied for the sorption removal of COD from real wastewater samples (efficiencies between 70% and 81%) and degradation of Methylene blue by Fenton reaction (degradation efficiencies up to 100%). The main conclusion from this work is that the best set of values depends on the target environmental application, and this set of values were determined for the two applications studied.

## 1. Introduction

In the last decade, substantial scientific literature has been published concerning ways to produce and apply nanomagnetic particles in broad fields of science and technology [1,2,3,4,5,6,7,8,9,10,11,12]. Many production methods exist and were proposed [13,14]. For environmental processes several have been applied, but when reaching real practical level, co-precipitation, and sometimes thermal decomposition, are the only methods that are economical and technologically viable [15].

The large majority of environmental applications [16,17,18] are usually concerned with the removal of contaminants/nutrients from water/wastewaters using nanomagnetic particles as sorption vehicles [19,20,21,22,23,24,25] that are at the end magnetically separated (containing the respective contaminant/nutrient) from the watery effluent and then recycled and reused. Another environmental technique is the degradation of contaminants present in watery systems [26,27,28,29,30,31,32,33] by the action of free radicals that appear due to heterogeneous (photo-)Fenton reaction that occurs when the surface of the iron-oxide nanomagnetic particles is in the presence of hydrogen peroxide (H_2_O_2_).

Nonetheless, many of the published articles do not study the application of the obtained particles to real industrial/treatment-plant water samples, and thus, their use in real systems is hard to evaluate. Furthermore, even less literature deals with the problematics of determining the relation between the final composition and characteristics of the nanomagnetic particles (and thus of the variables defined in the production process) and the efficiency of the environmental remediation.

In this work we report a study concerning the manufacturing of nanomagnetic particles by a reverse co-precipitation method, and the influence of the operational variables on the properties of the obtained particles. Then we determine the efficiency for each of the obtained different groups of particles, concerning environmental degradation/removal of MB/COD in water/real wastewater samples (from wastewater treatment plant of Salamanca, Spain), by using two technologies: magnetic sorption and Fenton process.

## 2. Materials and Methods

### 2.1. Magnetic Particle Manufacturing

Materials: We have used iron sulfate heptahydrate (FeSO_4_∙7H_2_O, 99%, Sigma, St. Louis, MO, USA), as the precursor salt. The alkaline bases used were sodium hydroxide and ammonium hydroxide (NaOH, 98%, Sigma-Aldrich, Madrid, Spain and NH_4_OH), the second, in a concentration between 28–30% NH_3_ (Sigma Aldrich, Madrid, Spain). As surfactants Tween 80 (polyoxyethylene (20) sorbitan monooleate, Merck, Madrid, Spain) and citric acid (C_6_H_8_O_7_, Sigma-Aldrich) were used. Distilled water was also used for the solutions. In this work it was used the following instrumentation: oven (Argolab G-TCF-120, Porto, Portugal); fridge (TEKA Cl3 350, Madrid, Spain); analytical balance (Sartorius CubisMSE225S-100-DA, Porto, Portugal); orbital shaker (ELMI Sky Line Shaker DOS-20 M, Porto, Portugal); magnetic separation system (home-made); Buchner filter device (Nahita 300 mL, Porto, Portugal), among other instrumentation.

Methods: We have chosen the reverse co-precipitation method [34,35,36,37,38] as the magnetic particle production method. This method, among other important characteristics, allows to maintain and easily control the pH value during all the process, in opposition to what occurs in the regular co-precipitation method. We have modified the process in order to be the most adequate for the real large-scale applications under study in order to maximize its short reaction time and production capability (necessary to treat high-throughputs like the ones presented at real environmental applications, and at the same time, decrease the costs of the process and produce low-cost particles—mandatory factor in environmental applications) [39]. The major modification in the process was to replace the inert atmosphere by air in order to obtain a high polydispersity, giving rise to particles with different sizes (ranging from micron to nanosized particles). The motive behind the use of this kind of mixture was to obtain a final product characteristic representing a mixture of the characteristics and efficiency of both sizes of particles (capable of treating large volumes of water flows and of being retained and recovered by moderate magnetic forces [15]—micron size—while maintaining a high level of efficiency concerning sorption and Fenton reaction—nanosize). In this study 4 factors (independent variables) were evaluated: the type of alkaline solution (NaOH or NH_4_OH), the type of surfactant (Tween 80, citric acid or none), the initial concentration of iron salt (0.2 or 0.4 M) and drying temperature. For these factors, 2 dependent properties of the particles were studied: the sizes of obtained crystallites and the magnetic susceptibility.

**Figure 1:**
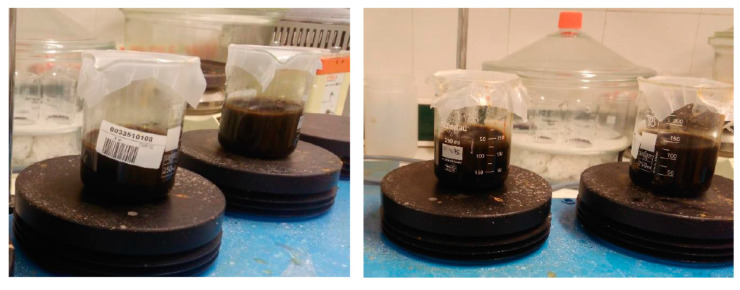
Reaction stage of the particle production process.

Experimental Procedure: 50 mL solutions of 0.2 and 0.4 M of FeSO_4_ were prepared with distilled water and with mechanical stirring for 10 min. The alkaline base solutions were carried out by mixing the base with equal parts of distilled water (1:1volume) to reach a volume of 100 mL and checking that the pH was greater than 12. To the alkaline-based solution, 10 mL of iron sulfate solution were slowly added over 10 min and, where appropriate, the surfactant was added. After addition, the reaction was allowed to proceed for 30 min. The reaction was carried out in a beaker with constant mechanical stirring and at controlled room temperature (23 °C). During this time, the pH was checked for no significant changes (always above 12) and the reaction vessels were capped to avoid evaporation of the alkaline base as much as possible (Figure 1). After some minutes, the stirring was stopped, and the magnetic particles formed were magnetically separated (the intensity of the applied magnetic field reached 0.05 T). Subsequently, successive washes were carried out in order to remove from the surface of the particles any remaining impurity resulting from the reaction. After the washes, the particles were centrifuged and then were dried in an oven to remove any moisture they may retained. Twelve different experiments (each repeated 3 times) were performed varying the previously mentioned factors as indicated in Table 1. In the case of the addition of surfactant, the difference of adding it before or after the mixing of the reagents was evaluated (in the case of citric acid, when added before iron sulfate, it reacted quickly with the base, lowering the pH and obtaining worse results, so it was later discarded). Tween 80 a or b refers to whether it was added before or after, respectively, and citric acid a or b, refers to the amount that was added (0.6 or 0.3 g).

Analytical methods: To characterize the particles obtained and evaluate the different factors, various studies were done. To verify that magnetite was obtained, X-ray studies were carried out, where the percentage of magnetite and the size of the crystallites were determined (equipment used: X-ray diffraction (XRD) Bruker D8 Advance, Karlsruhe, Germany). To assess the size of the particles and their morphology, SEM (JEOL JSM-840, Madrid, Spain) was performed. To determine magnetic susceptibility samples were analyzed with a Kappabridge KLY-2 susceptometer (Madrid, Spain) (a semiautomatic auto balance inductivity bridge); we have measured each sample a minimum of 3 times, operating at an alternating weak field of 4 × 10^−4^ T, 920 Hz and field intensity of 300 Am^−1^; the system has a high sensitivity resulting in a resolution of 4 × 10^−8^ SI and an accuracy of ±0.1% within one measuring range. The system was calibrated with an Etalon standard (1167 SI) with and accuracy of ±3%.

### 2.2. Sorption Experiments

Materials: The magnetic particles used where the ones prepared by the methods detailed in Section 2.1, while the wastewater samples were collected at the Salamanca Wastewater Treatment Plant (Salamanca, Spain). For analysis of the environmental parameters, we have used COD Merck Kits (Merck, Madrid, Spain). To carry out this analysis the following instrumentation was used: spectrophotometer (Merck Prove 300 Spectroquant^®^, Porto, Portugal), digestor (Merck Prove 300 Spectroquant^®^ Thermoreactor TR-420, Porto, Portugal); other instrumentation detailed in point 2.1.

Experimental Procedure: To evaluate the adsorption of organic matter contained in the wastewater samples by the produced magnetic particles, the procedure followed was: 1. Weighing of magnetic particles (100 mg); 2. Introduction of the particles in a glass bottle; then 5 mL of the wastewater sample was added; 3. The above mixture was shaken in the Orbital Shaker (SicLabs, Madrid, Spain) for 1 h; 4. The supernatant was recovered by magnetic separation; 5. Chemical oxygen demand (COD) of the supernatant was analyzed using the Merck kits.

### 2.3. Fenton Experiments

Materials: Regenerating solutions (according to [40,41], Hydrogen Peroxide (H_2_O_2_, Panreac, 30% v/v) and Methylene blue (Powder, Sigma-Aldrich, Madrid, Spain). The magnetic particles used were the ones prepared by the methods detailed in Section 2.1, while the samples were prepared by dissolution of methylene blue in water. In this work it was used the following instrumentation: Magnetic Thermostirrer (Nahita 690/1, Porto, Portugal), Spectrophotometer (DR 3900, VIS, Porto, Portugal), other instrumentation as detailed in point 2.1.

Experimental Procedure: We started by regenerating 60 mg of particles according to the process described in [40,41]. Then we wash them and insert them into a 20 mL solution containing 1 mL of 15% methylene blue and 0.5 mL of 30% H_2_O_2_. After this, we stirred the solution and measured COD with Merck kits, initially and after 72 h.

## 3. Results

### 3.1. Magnetic Particle Manufacturing

#### 3.1.1. XRD Analysis

**Figure 2:**
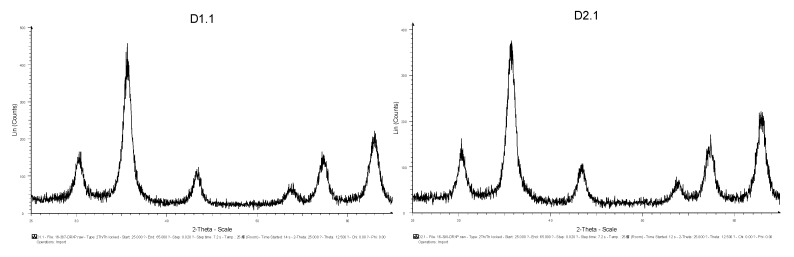

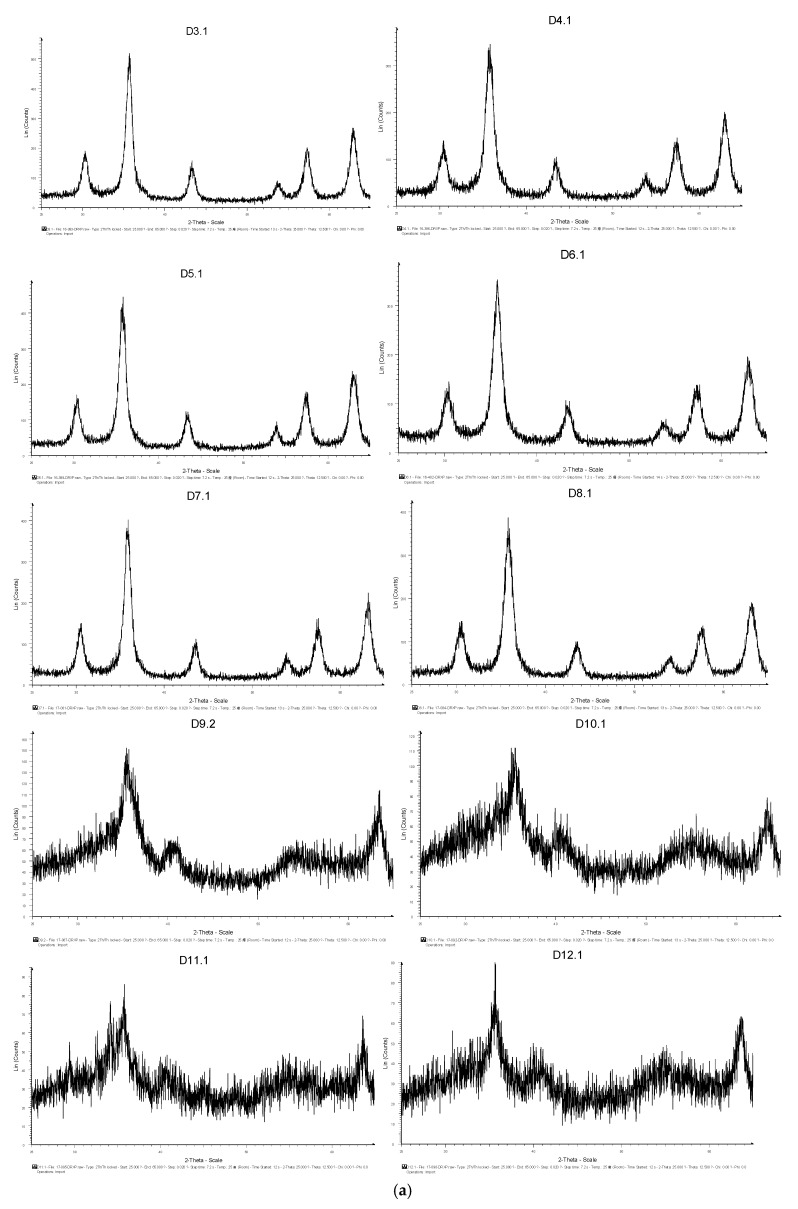

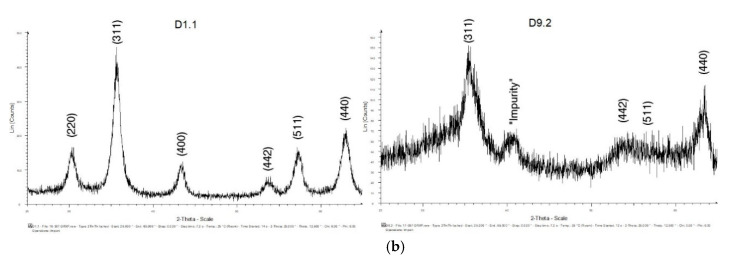
X-ray diffraction (XRD) diffractograms for the different particles produced (**a**) and typical peaks of magnetite assigned, as well as the peak corresponding to the “impurity” that appears in samples 9–12 (**b**).

The XRD diffractograms obtained for the produced particles are presented in Figure 2. The crystallite size obtained by applying Scherrer formula is presented in Table 2. The Scherrer formula was applied for the most intense peak (ca. 36°) after the measurement of a crystalline standard for line shape (NIST SRM 660c, LaB6) in the same measurement conditions. The instrumental contribution to the 36° peak width was calculated by carrying out a profile analysis of the standard diffraction pattern.

#### 3.1.2. Magnetic Susceptibility Determination

Magnetic susceptibility (χm) was measured in all samples to characterize their magnetic properties. The results are shown in Table 2.

#### 3.1.3. SEM Images

**Figure 3:**
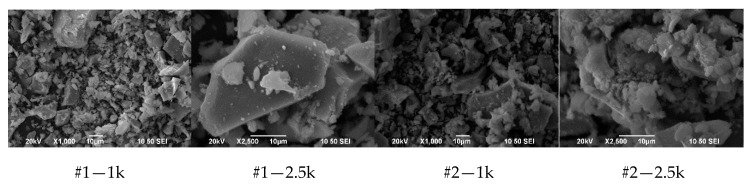

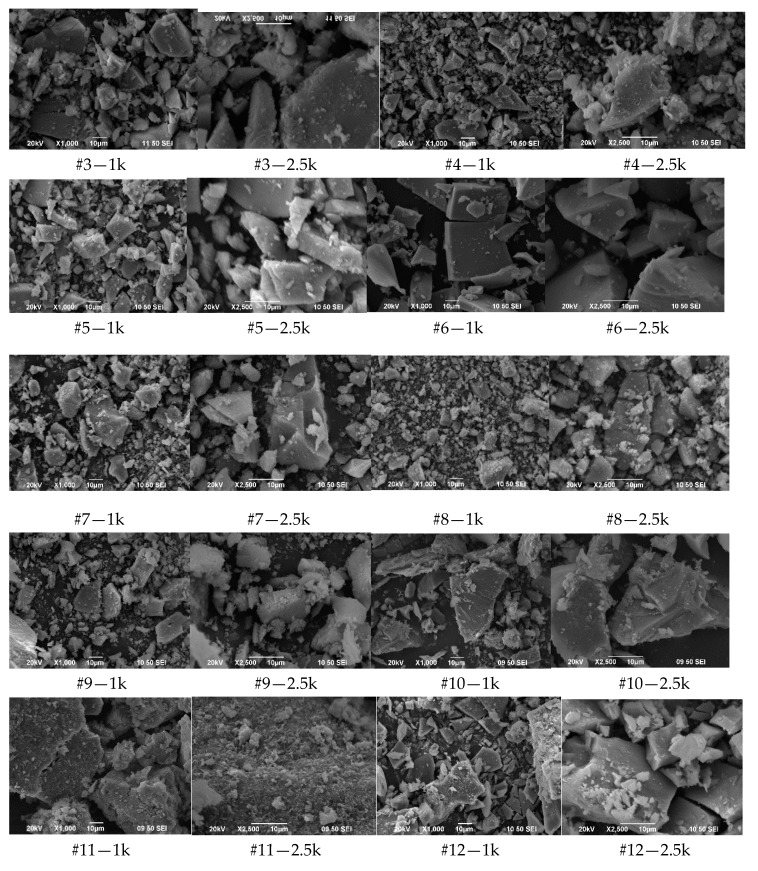
SEM images of the produced particles. #number is the number of the sample, 1 k represents a 1000× magnification, while 2.5 k represents a 2500× magnification.

SEM images were obtained for each sample to illustrate particle size distribution and morphology (Figure 3).

#### 3.1.4. Discussion of Results

XRD results indicate that magnetite was the crystalline phase in all samples. In fact, diffraction patterns were compatible with pure magnetite (formula/structure) except inexperiments 9 to 12, where a spurious peak around 2Theta = 41° arises (Figure 2). The crystallite size obtained was estimated by applying Scherrer formula using the highest intensity XRD peak, namely (311), and is presented in Table 2. Among all the samples, crystallite size ranges between 45 Å and 111 Å, with experiment 9 having the smallest average size and experiment 7 the largest. A qualitative inspection of diffraction patterns suggests that peaks were sharper in samples 1 to 8 and significantly broader in samples 9 to 12, with a drastic reduction of signal-to-background ratio in the second group. These facts correlate with a reduction (ca. 18%) of the mean crystallite size from 95 to 78 Å, respectively (Table 2).

It was seen that experiments 1–8 give higher values of magnetic susceptibilities while in experiments 9–12 the susceptibility value lowers, in some cases, by a factor of 2, which is in accordance with the presence of impurities and/or the reduction of crystallinity.

SEM images show no significant differences among the particle size and shape of different experiments, showing a complex mixture of sizes from tens of microns to nanosized particles. Interestingly, crystallite and particle size were very different in all cases. Scherrer crystallite-size refers to a coherently diffracting domain, while SEM particle size illustrates ‘grain’ size. Our results suggest that fabrication paths result into similar grain-size distribution, but the number of crystallites per grain increases in experiments 9 to 12. This seems to have a clear impact on magnetic properties, reducing magnetic susceptibility by two orders of magnitude (Table 2).

The role of different factors is discussed below:

- Factor 1: Alkaline base

To analyze the influence of the type of alkaline solution, experiments 1, 2 and 6 were compared with experiments 10, 11 and 12, respectively. The first three were made with NH_4_OH and the others with NaOH. On the other hand, during the separation of the particles, it was observed that the separation was much faster for NH_4_OH experiments than for NaOH experiments, and as they possess more or less the same size, it confirms the less pure (or shallow) character of the magnetite particles obtained when NaOH was used. While internal microstructure of grains (crystallite-size) could probably have an important role in this behavior, the presence of impurities needs to be further investigated in the future. It was evident that the use of NH_4_OH makes it easier to obtain magnetite particles with better magnetic characteristics (about 100 times higher values).

- Factor 2: Drying Temperature

To study this factor, experiments 1 and 2 were compared with experiments 5 and 7, respectively. The first ones were obtained at a drying temperature of 50 °C, while the second ones at 90 °C. As already seen in the X-ray results, all of these samples gave 100% magnetite. It can be seen that they do not differ much for the two temperatures studied. However, comparing the results of the magnetometry, it was observed that the particles obtained at a higher drying temperature have a higher magnetic susceptibility.

- Factor 3: Initial concentration of Fe_2_SO_4_∙7H_2_O

In experiments 1 and 8 the results were compared for an initial concentration of Fe_2_SO_4_∙7H_2_O equal to 0.2 and 0.4 M, respectively. The main difference observed was that a higher magnetic sensitivity was obtained for a higher concentration. However, no other comparisons could be made to study the relationship between this and other factors.

- Factor 4: Surfactant

The influence of the type of surfactant was studied by the following experiments:

(a) Without surfactant/Tween 80: experiments 1, 2 and 3;
(b) Without surfactant/Citric acid: experiments 1, 4 and 6;
(c) Tween 80/Citric acid: experiments 3 and 6;
(d) Without surfactant/Tween 80 (drying at 90 °C): experiments 5 and 7

From comparison (a) it was deduced that the use of Tween 80 allowed to obtain particles with better characteristics (greater magnetic susceptibility) and even better if this surfactant was added after having added the iron sulfate solution to the NH_4_OH and not before. From comparison (b) it was deduced that the use of citric acid in an amount of 0.3 g was better than using 0.6 g or not using surfactant. Finally, according to (c), comparing the results between both surfactants, it was seen that better results were obtained with Tween 80. However, from (d), it was observed that the results were better when performed without surfactant than with Tween 80, indicating a possible interaction between the use of Tween 80 and the drying temperature of the particles.

- Optimized values for production

Based on the results obtained, it was concluded that better results were reached when the base was NH_4_OH and the drying temperature was 90 °C. Increasing the concentration may also favor the magnetic susceptibility of the particles, but there were no data on the interaction this may cause with the other factors. Regarding surfactant, it was established that better results were obtained using Tween 80 instead of citric acid, but the influence of Tween 80 when compared with samples produced without surfactant was not clear. For these reasons, it was established that considering only particle characteristics, the operating conditions for the production of magnetic micro and nanoparticles in a scale-up plant would be 90 °C (363 K) drying temperature, NH_4_OH as alkaline base, initial concentration of FeSO_4_∙7H_2_O equal to 0.2 M and reaction without surfactant (conditions analogous to experiment 5).

### 3.2. Sorption Experiments

**Figure 4:**
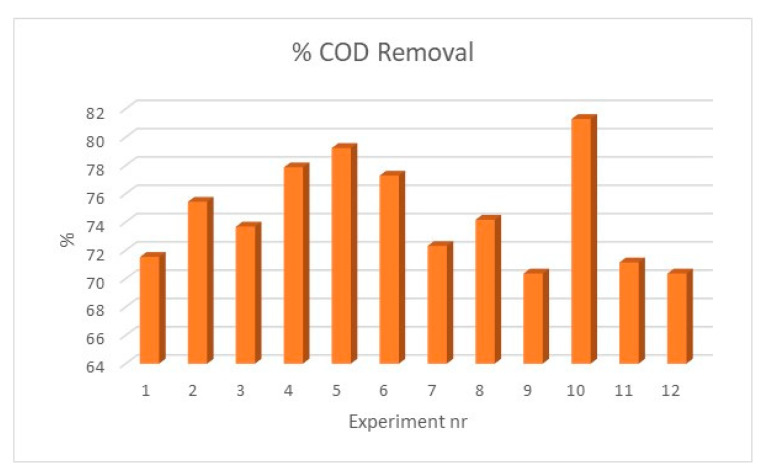
COD removal (%) for the wastewater samples for the particles produced in each experiment.

In Table 3 are presented the results obtained after sorption experiments to remove organic matter (measured as COD) from wastewater samples. Figure 4 reflects this result.

#### Discussion of Results

All the particles present a good efficiency for COD removal, especially if we notice that all results were obtained after only one hour of sorption. The values were between 70% and 81%. The best value was obtained in experiment 10 and the worst in experiments 9 and 12, curiously all obtained by using NaOH as alkaline base. Experiments with NH_4_OH seem more consistent and it was important to notice that again experiment 5 seems the most appropriate option (presenting the second-best removal efficiency, around 80%).

### 3.3. Fenton Experiments

**Figure 5:**
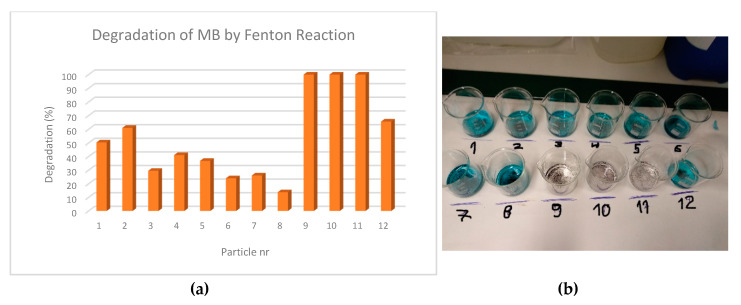
Degradation (%) of the colorant present in the water samples by Fenton reaction: (**a**) chart; (**b**) photograph of the final process results.

In Table 4 are presented the results obtained for the degradation of the samples containing the colorant, which are also shown in Figure 5.

#### Discussion of Results

It was clear that particles produced by experiments 9, 10 and 11 present excellent results (100% degradation). These represent the cases where they were obtained by NaOH alkaline base. Their good performance was probably due to the initial activation stage that was much more effective in this case than in the case of the magnetite particles obtained by NH_4_OH (this probably relates to the amount of available Fe at the surface of the particles that was more exposed in the particles obtained by the NaOH process). The other remaining particles behave quite differently; among these, particles from experiments 1, 2 and 12 stand out with efficiencies above 50%. For the case of Fenton reaction and degradation of colorant we would then choose particles generated by experiment 9, 10 or 11, and among these, would prefer particles from experiment 10 as they also behave superbly considering sorption experiments and do not require any surfactant for their production (the performance of the particles for Fenton reaction usually decreased when a surfactant was used in their manufacture). It is important to notice that higher magnetic fields must be applied in the cases of samples 9–12 to recover the particles, as their magnetic characteristics were lower than the ones presented by samples 1–8.

## 4. Conclusions

From the point of view of particle characteristics and environmental protection (less reagents) experiment five represents the best-case scenario (NH_4_OH as alkaline base, no surfactant required, concentration of salt of 0.2 M and drying temperature of 90 °C). All particles behave well concerning COD sorption efficiency from the real wastewater samples, but particles from experiment five behave better. However, from the point of view of degradation of methylene blue by Fenton reaction, particles produced in experiment 10 are preferable (NaOH as alkaline base, no surfactant required, concentration of salt 0.2 M and drying temperature of 50 °C).

## Figures and Tables

**Table 1:** Experiments and the values of the operational variables.

Experiment n.	Alkaline Base	Surfactant	FeSO_4_ (M)	Drying T (°C)
1	NH_4_OH	–	0.2	50
2	NH_4_OH	Tween 80 a	0.2	50
3	NH_4_OH	Tween 80 b	0.2	50
4	NH_4_OH	Citric acid a	0.2	50
5	NH_4_OH	–	0.2	90
6	NH_4_OH	Citric acid b	0.2	50
7	NH_4_OH	Tween 80 a	0.2	90
8	NH_4_OH	–	0.4	50
9	NaOH	–	0.2	50
10	NaOH	–	0.2	50
11	NaOH	Tween 80 b	0.2	50
12	NaOH	Citric acid b	0.2	50

**Table 2:** Crystallite sizes estimated by the Scherrer formula and corresponding magnetic susceptibility values per each experiment.

Experiment nr.	Crystallites Size (Å)	χ_m_ (×10^5^) (SI Units)
1	92.9	7507.7
2	92.4	10,485.2
3	98.3	10,643.4
4	85.7	8367.7
5	95.5	11,752.8
6	88.6	10,225.3
7	111.3	11,346.3
8	96.3	9060.1
9	45.3	116.0
10	87.0	63.8
11	90.7	123.6
12	71.0	141.8

**Table 3:** Initial and final chemical oxygen demand (COD) values and removal % by the sorption process.

Particles nr.	COD (mgO_2_/L)	Removal (%)
Initial	1026	–
1	292	71.5
2	252	75.4
3	270	73.7
4	227	77.9
5	213	79,2
6	233	77.3
7	284	72.3
8	265	74.2
9	304	70.4
10	192	81.3
11	296	71.2
12	304	70.4

**Table 4:** Results of the degradation of methylene blue in water by the Fenton process.

Particles nr.	Relative Absorbance	% Degradation
1	0.50	50.4
2	0.39	61.0
3	0.70	29.5
4	0.59	41.1
5	0.63	36.8
6	0.76	24.0
7	0.74	26.1
8	0.86	13.8
9	0.00	100.0
10	0.00	100.0
11	0.00	100.0
12	0.34	65.6
